# Resveratrol differentially modulates inflammatory responses of microglia and astrocytes

**DOI:** 10.1186/1742-2094-7-46

**Published:** 2010-08-17

**Authors:** Xiaofeng Lu, Lili Ma, Lingfei Ruan, Yan Kong, Haiwei Mou, Zhijie Zhang, Zhijun Wang, Ji Ming Wang, Yingying Le

**Affiliations:** 1 Key laboratory of Nutrition and Metabolism, Institute for Nutritional Sciences, Shanghai Institutes for Biological Sciences; Chinese Academy of Sciences, Shanghai 200031, China; 2 Graduate School, Chinese Academy of Sciences, Shanghai 200031, China; 3 Laboratory of Molecular Immunoregulation, Cancer and Inflammation Program, Center for Cancer Research, National Cancer Institute-Frederick, Frederick, Maryland, USA; 4 Shanghai Synchrotron Radiation Facility, Shanghai Institute of Applied Physics, Chinese Academy of Sciences, Shanghai, 201204, China

## Abstract

**Background:**

Inflammatory responses in the CNS mediated by activated glial cells play an important role in host-defense but are also involved in the development of neurodegenerative diseases. Resveratrol is a natural polyphenolic compound that has cardioprotective, anticancer and anti-inflammatory properties. We investigated the capacity of resveratrol to protect microglia and astrocyte from inflammatory insults and explored mechanisms underlying different inhibitory effects of resveratrol on microglia and astrocytes.

**Methods:**

A murine microglia cell line (N9), primary microglia, or astrocytes were stimulated by LPS with or without different concentrations of resveratrol. The expression and release of proinflammatory cytokines (TNF-α, IL-1β, IL-6, MCP-1) and iNOS/NO by the cells were measured by PCR/real-time PCR and ELISA, respectively. The phosphorylation of the MAP kinase superfamily was analyzed by western blotting, and activation of NF-κB and AP-1 was measured by luciferase reporter assay and/or electrophoretic mobility shift assay.

**Results:**

We found that LPS stimulated the expression of TNF-α, IL-1β, IL-6, MCP-1 and iNOS in murine microglia and astrocytes in which MAP kinases, NF-κB and AP-1 were differentially involved. Resveratrol inhibited LPS-induced expression and release of TNF-α, IL-6, MCP-1, and iNOS/NO in both cell types with more potency in microglia, and inhibited LPS-induced expression of IL-1β in microglia but not astrocytes. Resveratrol had no effect on LPS-stimulated phosphorylation of ERK1/2 and p38 in microglia and astrocytes, but slightly inhibited LPS-stimulated phosphorylation of JNK in astrocytes. Resveratrol inhibited LPS-induced NF-κB activation in both cell types, but inhibited AP-1 activation only in microglia.

**Conclusion:**

These results suggest that murine microglia and astrocytes produce proinflammatory cytokines and NO in response to LPS in a similar pattern with some differences in signaling molecules involved, and further suggest that resveratrol exerts anti-inflammatory effects in microglia and astrocytes by inhibiting different proinflammatory cytokines and key signaling molecules.

## Background

Microglia, the resident macrophage-like cells in the brain, play an important role in host defense and tissue repair in CNS [1,2]. Activated microglia produce a variety of pro-inflammatory mediators, including tumor necrosis factor α (TNF-α), interleukin-1β (IL-1β), IL-6, monocyte chemotactic protein 1 (MCP-1, CCL2), nitric oxide (NO), and reactive oxygen species (ROS). Activated microglia serve immune surveillance functions by removing foreign microorganisms, but may also result in excessive inflammatory responses in the CNS [1,2]. Astrocytes are the main glial cell type in the brain involved in maintaining CNS homeostasis. They also respond promptly to injury and regulate neuroinflammatory events [2-4]. Both in vitro and in vivo studies have documented the ability of astrocytes to produce a variety of cytokines, including IL-1, IL-6, IL-10, interferon-α (INF-α), IFN-β, TNF-α, TNF-β; and chemokines, including RANTES (CCL5), IL-8 (CXCL8) and MCP-1 [3]. Over-activation of glial cells and release of proinflammatory cytokines may lead to neuronal death [5-7], causing neuropathological changes in CNS diseases such as multiple sclerosis [8,9], Parkinson's disease [10,11], Alzheimer's disease [12] and AIDS dementia [13]. Therefore, limiting inflammatory cytokine production by activated microglia and astrocytes should be beneficial for prevention of neuroinflammation and neurodegeneration.

Resveratrol (3,4',5-trihydroxy-trans-stilbene) is a polyphenolic compound found in a large number of plant species that are components of human diet, including mulberries, peanuts, grapes and red wine. Accumulating evidence suggests that resveratrol may exert a protective effect in the CNS under pathological conditions, and that resveratrol is associated with reduced risks of cardiovascular disease, cancer, diabetes and AD [14-17]. Resveratrol has also been proposed to be an anti-inflammatory molecule [18]. In glial cells, resveratrol has been reported to inhibit LPS-induced production of NO and TNF-α by the murine microglia cell line N9 [19,20]; to inhibit prostaglandin E2 (PGE2) and free radical production by rat primary microglia [21], and to inhibit NO and PGE2 by the rat astroglial cell line C6 [22]. Microglia and astrocytes are two cell types with different biological characteristics and functions in the CNS, it is not clear if there are differences between these cells in response to LPS or if resveratrol inhibits the inflammatory responses of these cells to LPS through similar mechanisms.

In the present study, we first examined the expression of various proinflammatory cytokines (TNF-α, IL-1β, IL-6, MCP-1) and of iNOS by murine microglia and astrocytes in response to LPS, and the signaling molecules involved. We then determined the effects of resveratrol on microglial cell and astrocyte activation by LPS, and explored the underlying key signaling molecules.

## Methods

### Materials

Resveratrol, LPS and MTT were obtained from Sigma (St. Louis, MO). PD98059, SP600125, SB203580, sulfasalazine and curcumin were from Calbiochem (Darmstadt, Germany). Antibodies against both phosphorylated and unphosphorylated extracellular signal-regulated kinases (ERK1/2), p38, c-jun N-terminal kinase (JNK) were obtained from Cell Signaling Technology (New England Biolabs, Beverly, MA). Dual-Luciferase Reporter Assay System was from Promega Corporation (Woods Hollow Road, Madison, USA). LightShift Chemiluminescent EMSA kit was from Pierce (Pierce, Rockford, IL, USA). DMEM was purchased from Gibco BRL (Burlington, Ontario, Canada). Fetal bovine serum (FBS) was from Hyclone (Logan, UT). All other reagents were obtained from Sigma-Aldrich unless otherwise described.

### Glial cell cultures

**Figure 1:**
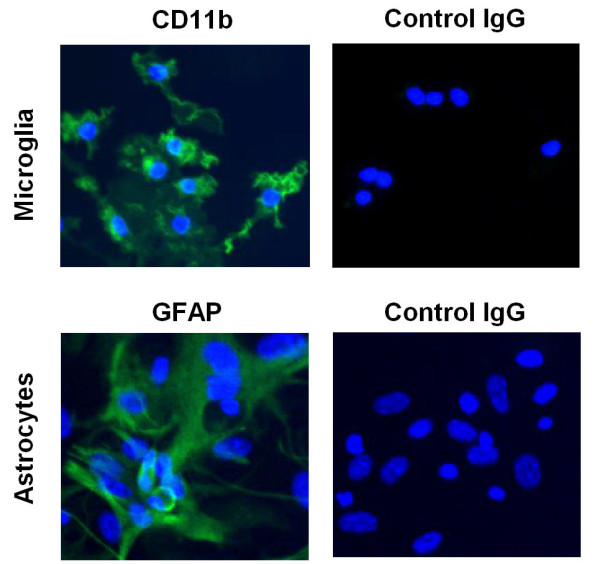
**Immunofluorescence staining of murine primary microglia and astrocytes with anti-CD11B and anti-GFAP antibodies, respectively**. Data are representative of three independent experiments with similar results.

Primary mouse microglia were isolated and purified from whole brain of newborn C57BL/6 mice as previously described [23]. Briefly, brain tissues were minced into small pieces. Cells were separated by trypsinization and cultured in 75 cm^2 ^tissue culture flasks with DMEM medium containing 10% FBS, 100 μmol/L non-essential amino acids, 5 μg/mL insulin, 100 U/mL penicillin and 100 μg/mL streptomycin. When cells grew to confluence (7-10 d), flasks were shaken overnight (200 rpm at 37°C) to loosen microglia and oligodendrocytes from the more adherent astrocytes. These less adherent cells were plated for 1 h and then lightly shaken to separate oligodendrocytes from the more adherent microglia. Microglia were seeded in cell culture plates for future use. After shaking to remove microglia and oligodendrocytes, the adherent astrocytes were purified by trypsinization, seeded in cell culture plates for 30 min, and removed adherent microglia. The purity of primary microglia and astrocytes is greater than 97% as determined by immunocytochemistry with antibodies against CD11b and glial fibrillary acidic protein (GFAP), respectively (Fig. 1). All experiments using mice were in accordance with the National Institutes of Heath Guide for the Care and Use of Laboratory Animals and were approved by the Biological Research Ethics Committee, Institute for Nutritional Sciences, Chinese Academy of Sciences. The murine microglial cell line N9 was a kind gift from P. Ricciardi-Castagnoli (Universita Degli Studi di Milano-Bicocca, Milan, Italy) [24]. The cells were grown in IMDM supplemented with 5% heat-inactivated FBS, 2 mmol/L glutamine, 100 U/mL penicillin, 100 μg/mL streptomycin, and 10 μmol/L 2-ME.

### Immunofluorescence staining

Murine primary microglia or astrocytes were cultured on slides in 24-well plates. The cells were fixed with 4% paraformaldehyde for 10 min at room temperature, washed with PBS, and then incubated with 5% BSA/PBS, 0.01% Tween-20 at room temperature for an additional 1 h. Rat anti-CD11b (1:25, BD Bioscience) or anti-GFAP (1:100, Zymed) antibody was applied to the slides and incubated overnight at 4°C. Rat IgG (Santa Cruz) was used as negative control. The slides were washed and incubated with FITC-conjugated goat anti-rat IgG (1:200) for 60 min, washed with PBS, stained with Hoechst 33342, and mounted. Immunofluorescence labeling was observed under a fluorescent microscope.

### RT-PCR and real-time PCR

Cells were cultured in medium without FBS for 24 h, then treated with 0.5 μg/mL LPS with or without various concentrations of resveratrol for another 8 h. Total RNA was extracted from cells with Trizol reagent (Invitrogen) and depleted of contaminating DNA with RNase-free DNase. cDNA was synthesized from 2 μg RNA with M-MuLV reverse transcriptase and random hexamer according to manufacturer's instructions (Fermentas, Burlington, Ontario, Canada). A total of 2 μL reverse transcription products was used for PCR. PCR products were visualized by ethedium bromide staining in 1.5% agarose gel and quantified using Gel-Pro Analyzer software (Media Cybernetics Inc., Silver Spring, Maryland, USA). Amplification of the target cDNA was normalized to β-actin expression. All experiments were replicated at least three times.

Quantitative real-time PCR was performed by using an ABI Prism 7500 sequence detector (Applied Biosystems Inc, Foster City, CA). Briefly, reverse-transcribed cDNA in duplicate samples were checked for cytokine or chemokine mRNA levels with SYBR Green PCR master kit (TOYOBO Biotech, Osaka, Japan) according to manufacture's instruction. The assays were initiated with 5 min at 95°C, and then 40 cycles of 15 s at 94°C, 1 min at 60°C. Amplification of the target cDNA was normalized to β-actin expression. Relative levels of target mRNA expression were calculated using the 2^-ΔΔC^T method. The primers for PCR and real-time PCR were listed in Table 1 and Table 2, respectively.

**Table 1:** PCR primers

Gene	PCR primers
TNFα	sense: 5'-ATGAGCACAGAAAGCATGATCCGCG
	antisense: 5'-CCCTTCACAGAGCAATGACTCCAAA
IL-1β	sense: 5'-ATGGCAACTGTTCCTGAACTCAACT
	antisense: 5'-AGGACAGGTATAGATTCTTTCCTTT
IL-6	sense: 5'-GATGCTACCAAACTGGATATAATC
	antisense: 5'-GGTCCTTAGCCACTCCTTCTGTG
MCP-1	sense: 5'-CTCACCTGCTGCTACTCATTC
	antisense: 5'-GCTTGAGGTGGTTGTGGAAAA
iNOS	sense: 5'-TGGGAATGGAGACTGTCCCAG
	antisense: 5'-GGGATCTGAATGTGATGTTTG
β-actin	sense: 5'-TGTGATGGTGGGAATGGGTCAG
	antisense: 5'-TTTGATGTCACGCACGATTTCC

**Table 2:** Real-time PCR primers

Gene	Real-time PCR primers
TNF-α	sense: 5'-AGCCGATGGGTTGTACCTTGTCTA
	antisense: 5'- TGAGATAGCAAATCGGCTGACGGT
IL-1β	sense: 5'-ACAGAATATCAACCAACAAGTGATATTCTC
	antisense: 5'-GATTCTTTCCTTTGAGGCCCA
IL-6	sense: 5'-ATCCAGTTGCCTTCTTGGGACTGA
	antisense: 5'-TAAGCCTCCGACTTGTGAAGTGGT
MCP-1	sense: 5'-CCCACTCACCTGCTGCTACT
	antisense: 5'-TCTGGACCCATTCCTTCTTG
iNOS	sense: 5'-GGCAGCCTGTGAGACCTTTG
	antisense: 5'-GAAGCGTTTCGGGATCTGAA
β-actin	sense: 5'-CAACGAGCGGTTCCGAT
	antisense; 5'-GCCACAGGATTCCATACCCA

### MTT assay

Cells cultured in 96-well cell culture plates were treated with various concentrations of resveratrol with or without 0.5 μg/ml LPS for 24 hours. Then the culture medium was removed and the cells were incubated with MTT (0.25 mg/ml) for 5 h at 37°C. The formazan crystals in the cells were solubilized with DMSO. The absorbance at 550 nm was determined by a microplate reader Multiskan JX (Themo LabSystems). Cell viability was expressed as a percentage of control.

### LDH assay

Cells were treated with different concentrations of resveratrol with or without 0.5 μg/ml LPS for 8 h. The supernatant was collected and LDH release was detected using a cytotox 96^® ^nonradioactive cytotoxicity assay kit (Promega) according to manufacture's instructions. Cell viability was expressed as a percentage of control.

### NO assay

Production of NO was determined by measuring the accumulated level of nitrite (an indicator of NO) in the supernatant after 24 h of LPS treatment with or without different concentrations of resveratrol using a colorimetric reaction with Griess reagent [25]. Briefly, 100 μL of supernatant were mixed with 100 μL Griess reagent [0.1% N-(1-naphthyl) ethylenediamine dihydrochloride, 1% sulfanilamide, and 2.5% H_3_PO_4_]. After incubation at room temperature in the dark for 10 min, total nitrites were measured spectrophotometrically at 540 nm. The concentration of nitrite in the sample was determined from a NaNO_2 _standard curve.

### Proinflammatory cytokine measurement by ELISA

Microglial cells (2 × 10^4^/well) or astrocytes (1 × 10^4^/well) were seeded into 48-well plates and cultured for 24 h. Cells were then washed twice with DPBS, and incubated in serum-free DMEM with or without different concentrations of resveratrol for 30 min followed by 0.5 μg/mL LPS for an additional 24 h. The supernatants were collected for measurement of TNFα, IL-6, and MCP-1; and cell lysate were made for detecting IL-1β; using ELISA as described by the manufacturer (Biosourse International).

### Western immunoblotting

N9 cells or murine primary astrocytes were grown in 60-mm dishes until subconfluency and then were cultured overnight in medium in the absence of FBS. The cells were pretreated with different concentrations of resveratrol for 1 h followed by LPS for different times (N9 cells: 30 min, astrocytes: 20 min), then were lysed with cold lysis buffer as described previously [26]. Cell lysate proteins were electrophoresed on a 10% SDS-PAGE gel, and transferred onto polyvinylidene difluoride membranes (Millipore Corporation, Bedford, MA). The membranes were blocked with 5% nonfat milk, and then were incubated with anti-phosphorylated ERK1/2, p38 or JNK antibody overnight at 4°C. After incubation with an HRP-conjugated secondary antibody, the protein bands were detected with a Supersignal West Pico chemiluminescenct substrate (Pierce, Rockford, IL) and X-Omat BT film (Eastman Kodak Company, Rochester, New York). For detection of total ERK1/2, p38, or JNK, the membranes were stripped with Restore Western Blot Stripping Buffer (Pierce, Rockford, IL), followed by incubation with specific antibodies. Immunoblot results were quantified using Gel-Pro Analyzer software (Media Cybernetics Inc., Silver Spring, Maryland, USA).

### Transient transfection and NF-κB luciferase reporter assay

One day before transfection, murine primary microglial cells (1 × 10^5^/well) or astrocytes (0.5 × 10^5^/well) were seeded into 24-well plates. Transient transfection of pNF-κB-Luc plasmid and control vector (a generous gift from Dr. J. Lu, Second Military Medical University, China) was performed using Lipofectamin 2000 according to the manufacture's recommendations. A pRL-TK-Renilla vector was used as an internal control for normalization of transfection and harvesting efficiency. The cells were cultured with transfection mixture for 5 h, and were then cultured in DMEM containing 10% FBS, 0.5 μg/mL LPS with or without different concentrations of resveratrol for 16 h. Luciferase activity of pNF-κB-Luc and pRL-TK constructs was measured sequentially using the Dual-Luciferase Reporter Assay System (Promega). Variation in transfection efficiency was normalized by dividing the promoter construct activity by the respective co-transfected pRL-TK luciferase activity. Promoter activity of the NF-κB was expressed in units relative to values measured in cells cultured with control medium.

### Nuclear extract preparation and electrophoretic mobility shift assay (EMSA)

Nuclear extracts were prepared as previously described [27]. Protein concentration was determined using a Bio-Rad protein assay kit with bovine serum albumin standards. Activation of AP-1 was assayed by EMSA using a LightShift Chemiluminescent EMSA kit (Pierce, Rockford, IL) according to the manufacture's instruction. Briefly, 6 μg of nuclear extract proteins were pre-incubated with binding buffer (50% Glycerol, 100 mmol/L MgCl_2_, 1% NP-40 and 1 μg/μL Poly (dI•dC)) for 5 min and then incubated with double-stranded biotin-labeled oligonucleotide containing consensus AP-1 binding site (5'-CGCTTGATGATGAGTCAGCCGGAA-3') for 15 min at room temperature. For competition experiments, unlabelled oligonucleotides were added to the nuclear extracts at a 200-fold molar excess before the addition of the biotin-labeled probe. DNA-protein complexes were analyzed by electrophoresis in 4% polyacrylamide gels. Complexes were transferred to a nylon membrane and crosslinked to the membrane using a hand-held UV lamp equipped with 312 nm bulbs. Migration of the biotinylated oligonucleotides and their complexes was detected by chemiluminescence followed by exposure of the membrane to X-ray films.

### Statistical analysis

Data are presented as mean ± SD. All experiments were performed at least three times. Data were analyzed by a 1-way or 2-way ANOVA with a post hoc Bonferroni test. Differences were considered significant at *p *< 0.05.

## Results

### LPS induces proinflammatory cytokine and iNOS expression in microglia and astrocytes in which different signaling molecules are involved

**Figure 2:**
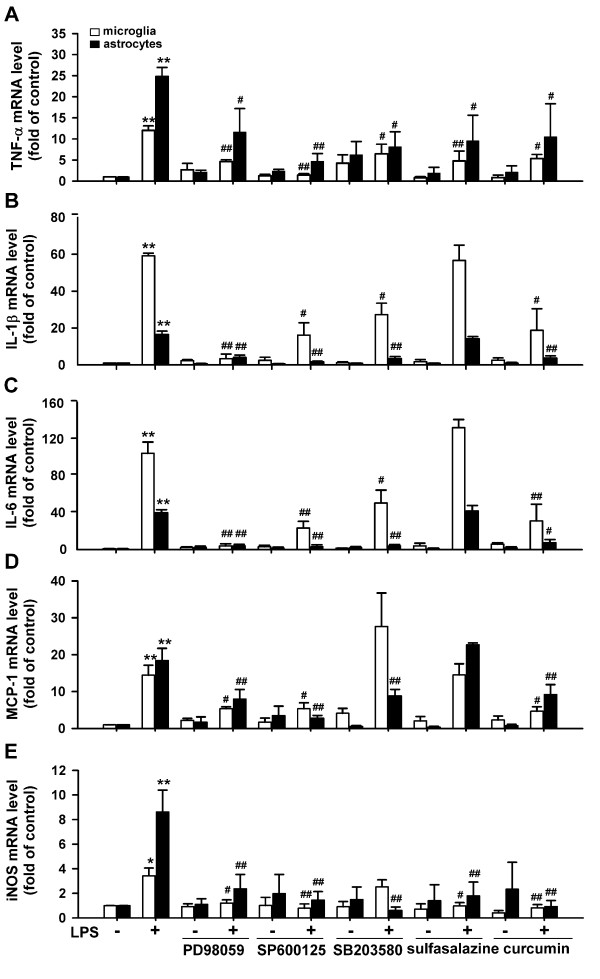
**Involvement of MAP kinases, NF-κB and AP-1 in LPS-induced proinflammatory cytokine and iNOS expression in glial cells**. Murine primary microglia or astrocytes pretreated with or without PD98059 (50 μmol/L), SP600125 (20 μmol/L), SB203580 (20 μmol/L), sulfasalazine (50 μmol/L), or curcumin (10 μmol/L) for 30 min were stimulated with 0.5 μg/mL LPS for 8 h. mRNA levels of proinflammatory cytokines and iNOS were examined by real-time PCR. Results are presented as mean ± SD for duplicate measurement from 6 independent experiments. *p < 0.05, **p < 0.01 compared with medium control; ^# ^p < 0.05, ^##^p < 0.01 compared with treatment with 0.5 μg/ml LPS.

We first examined the effect of LPS on proinflammatory cytokine and iNOS expression by murine primary microglia and astrocytes, and explored the signaling molecules involved. As shown in Fig.1, 0.5 μg/mL LPS significantly increased TNF-α, IL-1β, IL-6, MCP-1 and iNOS mRNA levels in both cell types. Activation of macrophages and microglia by LPS is thought to occur (primarily) by binding of LPS to Toll-like receptor 4, leading to activation of intracellular kinases (i.e., MAP kinases) and transcription factors like NF-κB and AP-1 [28]. We used inhibitors for MAP kinases and transcription factors to determine whether activation of ERK1/2, p38, JNK, NF-κB, or AP-1 contribute to the induction of proinflammatory cytokines and iNOS expression by LPS in microglia and astrocytes. Pretreatment of microglia and astrocytes with PD98059 (MEK inhibitor), SP600125 (JNK inhibitor), SB203580 (p38 inhibitor), sulfasalazine (NF-κB inhibitor) [29], or curcumin (AP-1 inhibitor) [30] significantly inhibited LPS-induced TNF-α expression in both microglia and astrocytes (Fig. 2A), suggesting the involvement of ERK1/2, JNK, p38, NF-κB and AP-1 in TNF-α expression. All these inhibitors, except for sulfasalazine, significantly inhibited LPS-induced IL-1β and IL-6 expression in both microglia and astrocytes (Fig. 2B and 2C), suggesting the involvement of ERK1/2, JNK, p38 and AP-1 but not NF-κB in IL-1β and IL-6 expression. While PD98059, SP600125, and curcumin inhibited MCP-1 and iNOS expression in both microglia and astrocytes, SB203580 inhibited MCP-1 and iNOS expression only in astrocytes, and sulfasalazine inhibited iNOS but not MCP-1 in both cell types, suggesting that ERK1/2, JNK and AP-1 are involved in LPS-induced expression of MCP-1 and iNOS in microglia and astrocytes, that p38 is involved in LPS-induced expression of MCP-1 and iNOS in astrocytes but not microglia, and that NF-κB is involved in LPS-induced expression of iNOS but not MCP-1 in both cell types. Taken together, ERK1/2, JNK and AP-1 are involved in LPS-induced TNF-α, IL-1β, IL-6, MCP-1 and iNOS expression in both microglia and astrocytes. p38 is involved in LPS-induced TNF-α, IL-1β, and IL-6 expression in both microglia and astrocytes but is involved in MCP-1 and iNOS expression in response to LPS only in astrocytes. NF-κB is involved only in LPS-induced TNF-α and iNOS expression in both cell types.

### Effects of resveratrol on cell viability and LPS-induced morphological changes in glial cells

**Figure 3:**
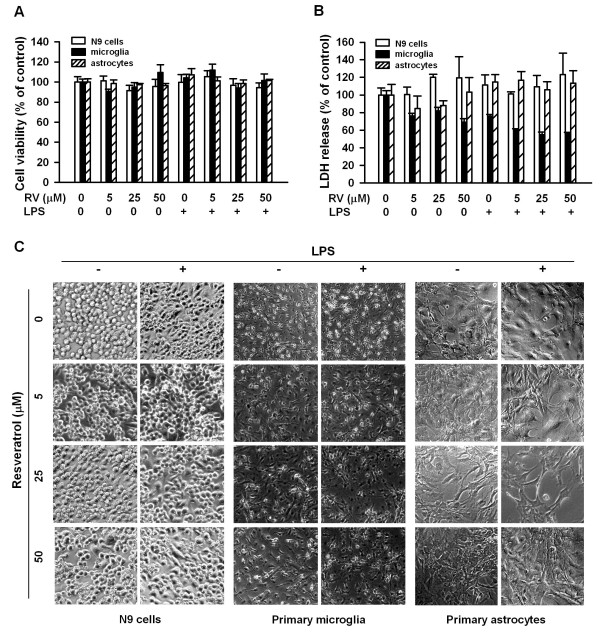
**Effects of resveratrol on glial cell viability and LPS-induced morphological changes**. (A-B), N9 cells, murine primary microglia, or astrocytes were treated with different concentrations of resveratrol with or without 0.5 μg/ml LPS. Cell viability was examined by MTT (24 h) and LDH (8 h) assays, respectively. The results are expressed as percentage of surviving cells over control cells. Data are presented as mean ± SD for three separate experiments. (C) N9 cells, murine primary microglia and astrocytes were treated with different concentrations of resveratrol with or without 0.5 μg/ml LPS for 8 h. Cell morphology was checked under microscope. Data are representative of three independent experiments with similar results.

At all concentrations used in this study, resveratrol alone or together with LPS did not show cytotoxicity to N9 cells, primary microglia and astrocytes as examined by MTT and LDH assays (Fig. 3A and 3B). Resveratrol treatment did not induce apoptotic cell death as examined by nuclear staining with Hoechst 33258 [31] (data not shown). A marked change in N9 and primary microglial morphology was observed at 8 h after stimulation with LPS (Fig. 3C): the LPS-stimulated microglial cells changing from an amoeboid shape to a multipolar (mostly bipolar, some tripolar) rod shape. LPS had no significant effect on astrocyte morphology. Resveratrol (5-50 μM) had no significant effect on microglial morphology induced by LPS (Fig. 3C). Resveratrol alone also had no significant effect on microglial cell and astrocyte morphology.

### Effects of resveratrol on pro-inflammatory cytokine gene expression and release

**Figure 4:**
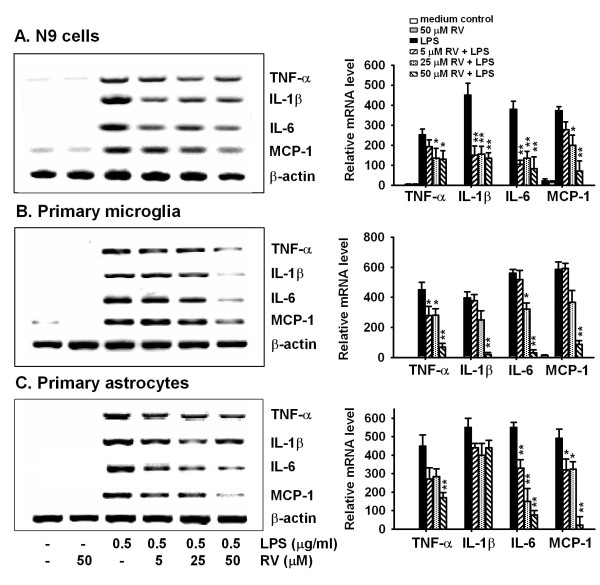
**Resveratrol inhibits LPS-induced cytokine mRNA expression in glial cells**. N9 cells (A), murine primary microglial cells (B), or astrocytes (C) were incubated with 0.5 μg/ml LPS in the presence or absence of different concentrations of resveratrol (RV) for 8 h. Cells were collected, and RNA was extracted to detect TNF-α, IL-1β, IL-6 and MCP-1 mRNA by RT-PCR. Data are presented as mean ± SD for three independent experiments. *P < 0.05, **P < 0.01 compared with treatment with LPS alone. Representative gels are shown.

**Figure 5:**
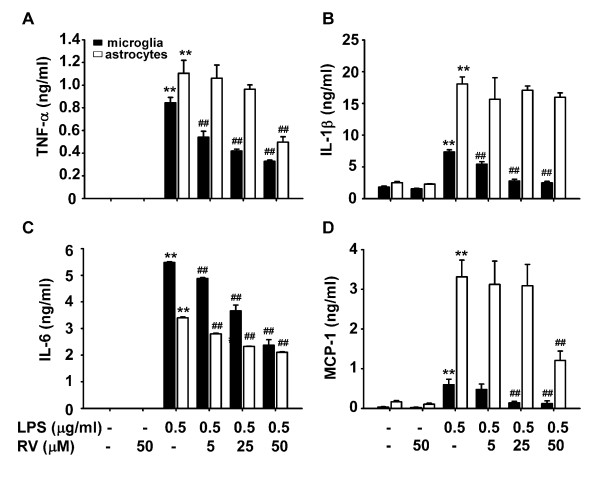
**Effect of resveratrol on LPS-induced cytokine production**. Murine primary microglial cells or astrocytes were treated with/without various concentrations of resveratrol (RV) in serum-free DMEM for 30 min followed by 0.5 μg/mL LPS for another 24 h. TNFα (A), IL-6 (C), MCP-1 (D) in supernatants and IL-1β (B) in cell lysate were detected by ELISA. Results are presented as mean ± SD for duplicate measurements from 3 independent experiments. **P < 0.01 compared with medium control or treatment with 50 μM RV alone. ^## ^P < 0.01 compared with treatment with 0.5 μg/ml LPS.

We next examined the effect of resveratrol on LPS-induced pro-inflammatory cytokine expression and release by microglia and astrocytes. As shown in Fig. 4, 0.5 μg/mL LPS significantly increased TNF-α, IL-1β, IL-6 and MCP-1 mRNA levels in cells of the microglial cell line N9, in primary microglia, and in astrocytes. Resveratrol inhibited LPS-induced cytokine mRNA expression in N9 cells and primary microglia (Fig. 4A and 4B). In primary astrocytes, resveratrol inhibited LPS-induced IL-6 mRNA expression in a dose-dependent manner and inhibited LPS-induced TNF-α and MCP-1 gene expression only at a high concentration (50 μmol/L), but had no effect on IL-1β gene expression (Fig. 4C). The effect of resveratrol on LPS-induced pro-inflammatory cytokine release by primary microglia and astrocytes was examined by ELISA. Murine primary microglia and astrocytes produced basal levels of IL-1β and MCP-1, but not TNF-α or IL-6 (Fig. 5). Stimulation of these cells with 0.5 μg/mL LPS significantly increased release of TNF-α, IL-6, and MCP-1; and production of IL-β (Fig. 5). Resveratrol dose-dependently inhibited LPS-induced TNF-α, IL-β, IL-6 and MCP-1 release by microglial cells. Resveratrol significantly inhibited LPS-induced release of TNF-α, IL-1β and IL-6 by primary microglia at concentrations as low as 5 μM. Resveratrol at tested concentrations (5-50 μmol/L) dose-dependently inhibited LPS-induced IL-6 release by primary astrocytes, including at the minimal concentration of 5 μM. Resveratrol also inhibited LPS-induced TNF-α and MCP-1 release, but only at high concentration (50 μmol/L), but had no effect on IL-1β production by primary astrocytes. Taken together, these results are consistent with the changes in cytokine mRNA levels (Fig. 4), and demonstrate that resveratrol differentially inhibits pro-inflammatory cytokine expression and release by LPS-activated microglia and astrocytes, with more potency in microglia.

### Effects of resveratrol on iNOS expression and NO production

**Figure 6:**
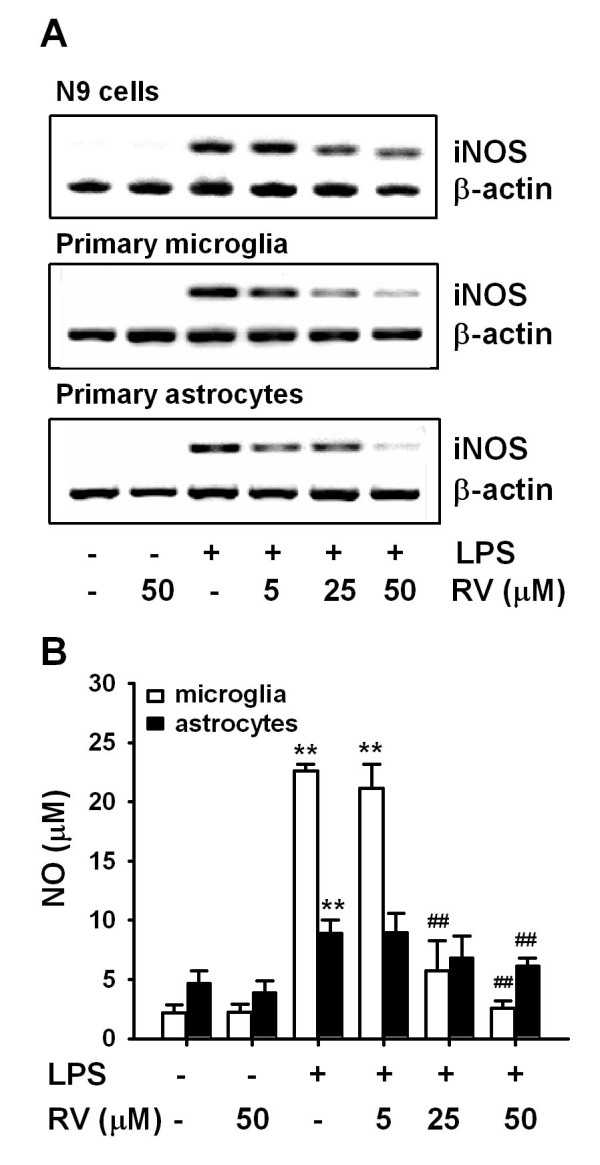
**Resveratrol inhibits LPS-stimulated iNOS mRNA expression and NO production**. N9 cells, murine primary microglial cells, or astrocytes were incubated with 0.5 μg/mL LPS in the presence or absence of different concentrations of resveratrol (RV). iNOS mRNA was examined by RT-PCR after 8 h incubation (A); NO in supernatant was measure by the Griess reaction after 24 h incubation (B). Results are presented as mean ± SD for 3 independent experiments. **P < 0.01 compared with medium control or treatment with 50 μM RV alone. ^## ^P < 0.01 compared with treatment with 0.5 μg/ml LPS.

We also examined the effect of resveratrol on LPS-induced iNOS expression and NO production by mouse N9 cells, primary microglia and astrocytes. LPS significantly stimulated iNOS gene expression in N9 cells, primary microglia and astrocytes. Resveratrol (5-50 μmol/L) dose-dependently inhibited LPS-induced iNOS gene expression in N9 cells and primary microglia. The expression of iNOS induced by LPS in astrocytes was inhibited by resveratrol only at a high concentration (50 μmol/L) (Fig. 6A). Consistent with its effect on iNOS mRNA, resveratrol significantly reduced NO production by LPS-stimulated primary microglia and astrocytes. While the inhibitory effect of resveratrol on primary microglia was dose dependent (Fig. 6B), with maximum inhibition shown at 50 μmol/L, only a high concentration of resveratrol (50 μmol/L) inhibited LPS-induced NO production in primary astrocytes. These results indicate that resveratrol is more potent in inhibiting LPS-induced iNOS expression and NO production by microglia than by astrocytes. It has been reported that LPS potently induces NO release in microglia cells by de novo synthesis of iNOS [24]. Thus, the effect of resveratrol on LPS-induced NO release may be due to the reduction of iNOS expression.

### Effect of resveratrol on MAP kinase activation by LPS

**Figure 7:**
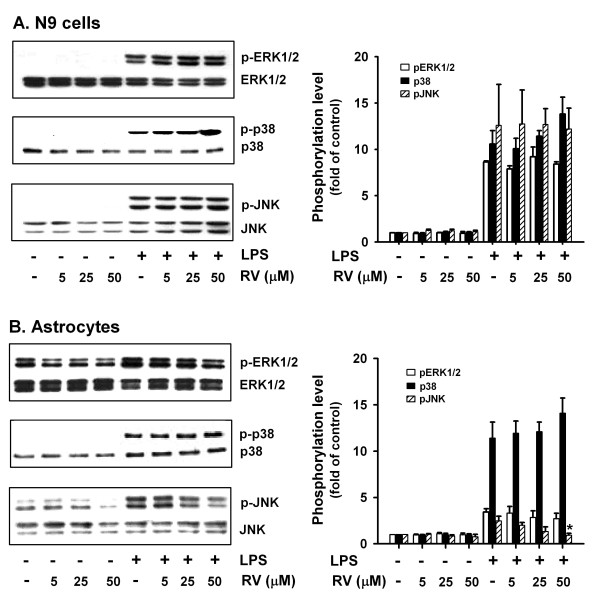
**Effect of resveratrol on MAP kinase activation by LPS in glial cells**. N9 cells (A) or murine primary astrocytes (B) were treated with various concentrations of resveratrol (RV) for 1 h followed by LPS stimulation (N9 cells: 0.2 μg/ml for 30 min; astrocytes: 0.5 μg/ml for 20 min). ERK1/2, p38, and JNK phosphorylation were examined by western blotting. Data are presented as mean ± SD for three independent experiments. Representative gels are shown.

Since the above results showed that MAP kinases are differentially involved in LPS-induced pro-inflammatory cytokine expression in microglia and astrocytes, we next investigated the ability of resveratrol to interfere with phosphorylation of MAP kinases in response to LPS. As N9 cells and primary microglia responded to LPS and resveratrol with similar patterns (Fig. 4) and the number of microglia that can be cultured from neonatal mice is limited, N9 cells were used in this and the following mechanistic studies. LPS significantly induced phosphorylation of ERK1/2, p38 and JNK in both N9 microglial cells and primary astrocytes. Basal level phosphorylation of ERK1/2 and JNK was detected in astrocytes. Resveratrol at a high concentration (50 μmol/L) slightly inhibited LPS-induced JNK phosphorylation in astrocytes but not in N9 cells, and had no effect on LPS-induced phosphorylation of ERK1/2 and p38 in either cell type (Fig. 7). These results suggest that, among MAP kinases activated by LPS, resveratrol is capable of inhibiting JNK activation in astrocytes. Thus, the inhibitory effect of resveratrol on LPS-induced proinflammatory cytokine expression and release may be mainly mediated by signaling molecules downstream of MAP kinases.

### Effects of resveratrol on NF-κB and AP-1 activation by LPS

**Figure 8:**
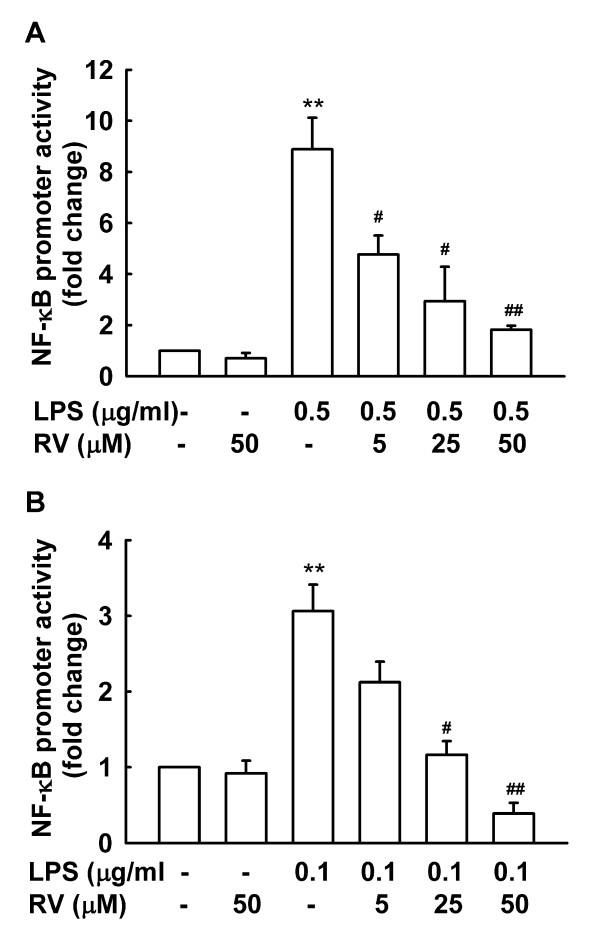
**Resveratrol inhibits activation of NF-κB by LPS**. Murine primary microglial cells (A) or astrocytes (B) transfected with an NF-κB luciferase reporter plasmid and a pRL-TK-Renilla vector were treated with LPS and different concentrations of resveratrol (RV) for 16 h. Cell lysates were prepared and luciferase activities were measured. Results are presented as mean ± SD for 3 independent experiments. **p < 0.01 compared with medium control or treatment with 50 μM RV alone. ^#^p < 0.05, ^##^p < 0.01 compared with treatment with LPS.

**Figure 9:**
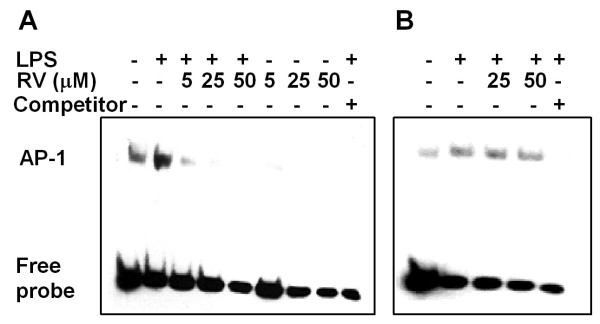
**Effect of resveratrol on LPS-induced AP-1 activation**. N9 cells (*A*) or murine primary astrocytes (*B*), pretreated with different concentrations of resveratrol (RV) for 30 min, were stimulated with 0.5 μg/mL LPS for 4 h. DNA-binding activity of AP-1 in the nuclear extract was examined by EMSA using a biotin-labeled AP-1 consensus probe. A 200-fold molar excess of cold probe was used to confirm the specificity. Data are representative of 3 separate experiments with similar results.

NF-κB and AP-1 are implicated in regulation of the expression of pro-inflammatory cytokines and iNOS by LPS [32]. Our results show that NF-κB and AP-1 play different roles in LPS-induced proinflammatory cytokine and iNOS expression in microglia and astrocytes (Fig. 2). The effect of resveratrol on activation of NF-κB and AP-1 by LPS was examined by luciferase reporter assay and EMSA, respectively. Luciferase reporter assay analysis showed that LPS significantly induces NF-κB activation in primary microglia (Fig. 8A) and astrocytes (Fig. 8B). Resveratrol dose-dependently suppressed LPS-induced NF-κB activation in both cell types. After LPS stimulation, DNA binding activity of nuclear extracts to AP-1 consensus oligonucleotides was significantly increased in N9 cells (Fig. 9A) but only slightly increased in astrocytes (Fig. 9B). Binding specificity was verified by incubating nuclear extracts from LPS-stimulated cells with excess unlabeled specific oligonucleotide probe (Fig. 9A and B, the last lane). Treatment with resveratrol alone inhibited basal binding activity of AP-1 in N9 cells (Fig, 9A). The increased AP-1 binding activity induced by LPS was inhibited in resveratrol-pretreated N9 cells but not in astrocytes (Fig. 9A and 9B). These results provide a mechanistic basis for the capacity of resveratrol to differentially inhibit LPS-induced microglia and astrocyte activation at the levels of NF-κB and AP-1.

## Discussion

The present study demonstrates that murine microglia and astrocytes produce proinflammatory cytokines and NO in response to LPS in a similar pattern with some differences in the signaling molecules involved. Resveratrol limited LPS-stimulated microglia and astrocyte activation with different potencies and through inhibiting different signaling molecules. To our knowledge, this is the first report of a difference between microglial cell and astrocyte in response to LPS, and of a difference in the capacity of resveratrol to protect microglia and astrocytes from inflammatory insults.

Microglia are the main resident immuno-competent and phagocytic cells in CNS. Astrocytes also play an important role in regulating inflammation in the CNS. LPS is capable of inducing production of pro-inflammatory cytokines and NO in both microglia and astrocytes [23,33,34]. Our study shows that LPS significantly induces the expression and production of pro-inflammatory cytokines (TNF-α, IL-1β, IL-6, and MCP-1), and enhances the expression of iNOS and production of NO, by primary microglia and primary astrocytes. N9 cells expressed pro-inflammatory cytokines and iNOs at the mRNA level in response to LPS in a pattern similar to that of primary microglia. These results indicate that the inflammatory responses of microglial cell and astrocyte to LPS are similar.

By binding to TLR4, LPS activates NF-κB through TAK1, and activates AP-1 through the TAK1-MAP kinase (ERK1/2, p38, JNK) pathway. NF-κB and AP-1 control inflammatory responses through the induction of inflammatory cytokines [28,32]. It has been reported that, in microglia, LPS stimulates TNF-α expression through activation of ERK1/2, p38, JNK/AP-1 and NF-κB [34-36]; stimulates IL-6 and MCP-1 expression through JNK2 and AP-1; stimulates IL-6 expression through p38 [34,37]; and stimulates iNOS expression through ERK1/2, p38 and NF-κB [35,38]. Our data demonstrate that, in addition to these mechanisms, LPS stimulates IL-1β expression through activation of ERK1/2, JNK, p38 and AP-1; stimulates IL-6 and MCP-1 expression through ERK1/2; and stimulates iNOS expression through JNK and AP-1. Taken together, these data suggest that MAP kinases, NF-κB and AP-1 are differentially involved in the production of proinflammatory cytokines and iNOS in microglia in response to LPS.

There are only a few reports regarding the involvement of MAP kinases and transcription factors in LPS-induced expression of inflammatory mediators in astrocytes. Treatment of astrocytes with LPS alone induces iNOS expression through ERK1/2- and NF-κB-related signaling pathways [39]. A combination of LPS and IFN-γ results in TNF-α and iNOS expression through activation of ERK1/2, p38 and JNK [39,40]. Our present study shows that LPS significantly induces ERK1/2, p38, and JNK phosphorylation and NF-κB activation but only slightly activates AP-1 in astrocytes, and that LPS induces proinflammatory cytokine (TNF-α, IL-1β, IL-6 and MCP-1) and iNOS expression in astrocytes through activation of ERK1/2, p38, JNK and AP-1. NF-κB is only involved in LPS-induced TNF-α and iNOS expression in astrocytes. Comparison of the results for microglia with those for astrocytes shows that similar signaling molecules (MAP kinases, NF-κB and AP-1) are involved in LPS-induced TNF-α, IL-1β and IL-6 expression, except that p38 is involved in MCP-1 and iNOS expression only in astrocytes and not in microglia. This may be due to differences in the biological characteristics of these two cell types.

Resveratrol has been reported to inhibit LPS-induced NO and PGE_2 _production by rat astroglioma cells [22], and to inhibit TNF-α, iNOS expression and NO production by a mouse microglial cell line [19,20]. Our results show that, in addition to inhibiting LPS-stimulated TNF-α and NO production, resveratrol also inhibits LPS-induced expression and production of IL-1β, IL-6, and MCP-1 in primary microglia and in the microglial cell line N9 (Fig. 4 and 5). At the tested concentrations (5-50 μM), resveratrol significantly inhibited LPS-induced proinflammatory cytokine production by primary microglia (TNF-α, IL-1β and IL-6) and astrocytes (IL-6), including significant inhibition at the lowest concentration of 5 μM. Furthermore, our results suggest that resveratrol differentially regulates the production of pro-inflammatory molecules and NO by microglia relative to astrocytes. Resveratrol dose-dependently inhibited the production of NO, TNF-α, IL-6 and MCP-1 by primary microglia in response to LPS, but only inhibited NO, TNF-α and MCP-1 production at a high concentration (50 μmol/L) and had no effect on IL-1β production in astrocytes (Fig. 5). Therefore, resveratrol has a more potent suppressive effect on the production of pro-inflammatory molecules by LPS-activated microglia.

Existing observations from both in vitro and in vivo studies have demonstrated that resveratrol has differential effects on MAP kinases and can inhibit the activation of NF-κB and/or AP-1 in a cell or tissue-specific manner [41,42]. Bi et al. [20] reported that resveratrol treatment (48 h) of LPS-stimulated (1 μg/mL) N9 cells inhibited LPS-induced p38 phosphorylation. Our study shows that LPS activates p38 in microglia and astrocytes, but we found that resveratrol has no effect on p38 phosphorylation in these cells (Fig. 7). The discrepancy may be due to differences in cell origin and experimental conditions. As our studies show that resveratrol has no effect on LPS-induced phosphorylation of ERK1/2 in either microglia or astrocytes, and only slightly inhibits LPS-induced JNK phosphorylation in astrocytes, we then examined the effect of resveratrol on signaling molecules downstream of MAPKs. NF-κB is a common regulatory element in the promoter region of many pro-inflammatory cytokines. Our studies show that resveratrol attenuates LPS-stimulated NF-κB activation in murine primary microglia and astrocytes. Consistently, other researchers have reported that resveratrol can suppress LPS-induced degradation of IκBα in the microglial cell line N9 [20], and can suppress nuclear translocation and activation of NF-κB in rat C6 astroglioma cells [22]. Resveratrol is an activator of SIRT1, which has been reported to inhibit NF-κB activity through deacetylation of the RelA/p65subunit of NF-κB [43]. The inhibition of SIRT1 signalingby LPS is partially responsible for the activation of NF-κB pathways and subsequent generation of TNF-α in Kupffer cells and macrophages [44]. Therefore, it should be quite interesting to investigate whether activation of SIRT1 signaling also contributes to the inhibitory effect of resveratrol on NF-κB activation by LPS in glial cells. Recent studies have shown that resveratrol inhibits LPS-induced NF-κB activation by targeting TANK-binding kinase 1 and RIP1 in the TRIF complex in a murine macrophage cell line [45]. Whether the inhibitory effect of resveratrol on LPS-induced NF-κB activation in microglia and astrocytes is mediated by a similar mechanism as that in macrophages will require further investigation. In addition to NF-κB, AP-1 has also beenshown to be involved in inflammatory responses in responseto LPS. Our results show that AP-1 is involved in LPS-induced IL-1β expression and release by microglia and astrocytes. Resveratrol inhibits LPS-induced AP-1 activation in microglia but not astrocytes, which may explain why resveratrol inhibits IL-1β expression and release by microglia but not by astrocytes. AP-1 is formed by dimerization of Jun proteins (c-Jun, JunB and JunD) or heterodimerization of a Jun protein with a Fos protein (c-Fos, FosB, Fra-1 and Fra-2) [46], and resveratrol has been reported to inhibit c-fos mRNA expression and AP-1 DNA binding in mouse skin [47]. We found that resveratrol has no effect on LPS-induced JNK phosphorylation but inhibits LPS-induced AP-1 activation in microglia. Contrarily, resveratrol slightly inhibits LPS-induced phosphorylation of JNK but has no effect on AP-1 activation by LPS in astrocytes. The contrary effect of resveratrol on JNK phosphorylation and AP-1 activation in microglia and astrocytes may be due to involvement of AP-1 components other than c-Jun in LPS-induced AP-1 activation in microglia, and LPS may activate different AP-1 components in microglia and astrocytes, but these possibilities need further investigation. Collectively, the different effects of resveratrol on proinflammatory cytokine and iNOS expression in response to LPS in microglia and astrocytes may be due to different effects of resveratrol on NF-κB and AP-1 activation in these two cell types. In addition, differences in biological characteristics of microglia and astrocytes may also contribute to their unique response to LPS and resveratrol.

Microglia and astrocytes play important roles in host defense during brain infection and inflammation. These cells produce pro-inflammatory mediators in response to pathologic stimuli such as LPS. As a potent source of proinflammatory cytokines and chemokines, microglia and astrocytes are pivotal in the progression of CNS diseases including Alzheimer's disease [12], multiple sclerosis [8,9], Parkinson's disease [10,11], and brain injury. Therefore, a therapeutic approach aimed at suppressing activation of microglia and astrocytes may alleviate inflammation in the CNS and thus retard the progression of these diseases. Our results suggest that the extent of inflammatory responses induced by LPS in microglia and astrocytes could be limited by resveratrol, with different potencies. Therefore, resveratrol is a natural product with therapeutic potential against CNS diseases involving overproduction of pro-inflammatory cytokines and NO.

## Competing interests

The authors declare that they have no competing interests.

## Authors' contributions

XL, LM, LR, YK HM, ZZ and ZW performed the experiments and analyzed the data. JMW provided useful advice and reviewed the manuscript. YL conceived the study, participated in its design and coordination, and wrote the manuscript. All authors read and approved the final manuscript.
